# DFMA-oriented modular and parametric design and secondary splitting of vertical PC components

**DOI:** 10.1038/s41598-023-30192-z

**Published:** 2023-03-01

**Authors:** Daode Dong, Yiquan Zou, Han Pan, Guowei Zhou, Yu Feng, Yuchen Tang

**Affiliations:** 1 School of Civil Engineering, Architecture and Environment, Hubei University of Technology, Wuhan, 430070 China; 2 China State Construction Engineering, 28 Nanli Road, Hongshan District, Wuhan City, 430000 Hubei Province China

**Keywords:** Civil engineering, Software

## Abstract

The design and production of assembled buildings are difficult to standardise, which limits their extensive application. In this paper, the design for manufacture and assembly (DFMA) concept is applied to the design of vertical precast concrete (PC) components for assembled buildings. Taking vertical PC components as an example, the rapid modelling and whole life-cycle application of DFMA-oriented PC components for assembled buildings are investigated. Firstly, the modular design method is introduced. By combining parametric design and building information modelling (BIM), we propose a modular, parametric design method and process of PC components based on DFMA. Secondly, the design of standard modules after secondary splitting is introduced for a better DFMA-oriented parametric design of PC components. We explored the secondary development process of DFMA and BIM and the module creation process of the parametric standard based on family templates and DFMA. Thirdly, based on the parametric standard module, the application of the optimised whole life cycle of design, manufacture and assembly of PC components is introduced. Finally, the feasibility of this method is verified by practical cases. The design process for PC components is fast, accurate and standardised. The integrated application of BIM is accelerated, and digital collaboration in assembly construction is strengthened. The study results are conducive to establishing a standardised design and production system for PC components, reducing design costs, improving design efficiency and the comprehensive benefits of assembled buildings.

## Introduction

In recent years, assembled buildings with prefabricated components as the core have been developing rapidly. Assembled buildings are energy-saving and environmentally friendly and embody the advantages of a short construction period and low labor cost^1–4^. They are an optimal choice for construction industrialization and residential industrialization. However, the current level of standardisation of their design and production is low, making it difficult to meet the requirements of industrialised design, production and assembly. It is therefore worthwhile to investigate the design and manufacturing process of assembled buildings. The key to the progression of assembled buildings lies in the construction of a standardised design and production system and the expansion of the application scope of standardised components^5^. For assembled buildings, integrated and modular building components need to be developed, and the production and construction need intelligent upgrading. The comprehensive benefits of assembled buildings should be improved to achieve the standardisation of design and production^6,7^.

This study focuses on assembled buildings, i.e. buildings consisting of fa ctory-made (prefabricated) components that are transferred and assembled on site. PC components are an important part of prefabricated buildings. They are used in the design, production, transport and assembly of assembled buildings^6^. Modular buildings based on PC components attract more and more attention from the architecture, engineering and construction (AEC) industry and are a new solution to the drawbacks of non-assembled buildings^7^. The modules used for construction are processed in the factory and later transported to the construction site for positioning and assembly^8^. Modular buildings can improve construction safety and efficiency. Nowadays, modular design is also a trend. With residential industrialization and building industrialization^9,10^, a module library is created based on the modular design theory. The relevant modules are selected from the module library and will be reorganised into new assembled buildings according to a certain logic to obtain design results and cope with the diversity of the design^11^. However, assembly buildings are currently modularized by being split into PC components such as beams and wall panels. There is no further splitting of PC components, which will cause design changes and inflexibility in factory production^12^. Therefore, it is important to determine the best modular design for the PC elements. The standardisation of PC components in design, production and assembly is considered at an early stage of design so that they can be used effectively and efficiently.

The design phase is important since it determines up to 80% of the operating costs of an assembled building^13^. A good design system is crucial to the production and installation of prefabricated buildings^14^. The design process of PC components still has many problems, which affect its standardization as well as the standardization of subsequent production and installation. Assembled buildings with different functions and shapes should be standardized in a unified manner. Usually, the design process for PC components is completed with uneven quality, inconsistent standards and a lack of effective framing, pacing and avoidance rules. The cost of design and management is high. These problems cannot be easily solved^15^. The component factory obtains the plans of the components for production, which often results in difficult splitting and unreasonable structural design. The design of components cannot be standardised, resulting in the production of components that cannot be productized^16^.

When using traditional CAD for component design, a series of problems can easily occur in different professions. For example, the design workload is high, the checks are cumbersome and the data transfer is poor. The production, transportability and installability of the components are not considered. The CAD drawings of the components can only guide their machining and installation. A great deal of on-site work needs to be carried out to ensure the quality of the building. Auxiliary design based on BIM technology makes up for the shortcomings of the 2D design platform^17^. By enhancing the links between the design drawings using its 3D visualisation features, the accuracy of the drawings is substantially improved^18^. Problems that only arise in the construction phase are brought forward to the design phase, eliminating risks in advance and increasing the workload in the design phase.

The construction industry is also learning from the manufacturing industry to improve the PC component design process. Design for manufacture and assembly (DFMA) has been introduced into the manufacturing industry; the requirements of manufacturing and assembly are considered during the design, making the product highly manufacturable and assembleable^19,20^. Manufacturing and assembly quality problems at a later stage of product development are fundamentally solved, thus reducing product design modifications and product costs, shortening product development cycles and improving product quality^21–24^. In recent years, with the reduction of on-site construction, off-site processing and manufacturing opportunities for DFMA are increasing.

The product design concept, DFMA, is introduced into the design process of components. DFMA mainly consists of design for assembly (DFA) and design for manufacturing (DFM). DFM refers to a product design meeting the requirement of good manufacturability so that products can be manufactured at the lowest cost in the shortest time with the highest quality^17,24,25^. DFA refers to a product design meeting the requirement of good assemblability, in other words, to achieve efficient assembly. Common methods include simplifying product design, reducing the number of components and using standard components^23^. Since the design process of DFMA considers the needs of production and assembly, it has been recognized and used in the construction industry, where standardisation and modularity become its key principles^24^. Researchers used this design method in construction to save time and reduce costs. Fox et al. proposed a strategy for the application of DFM to buildings^26^. Yuan et al. integrated BIM and DFMA to develop parametric concepts and processes for DFMA^14^. Arashpour et al.^27^ elaborated guidelines for DFMA in modular prefabrication of complex façade systems. Chen et al.^28^ highlighted the role of DFMA in curtain wall projects through a case system. In addition, several industrial reports have contributed to the popularization of DFMA in construction^29^.

The design of PC components based on DFMA cannot be achieved without digital design technology and design platforms^30^. Traditional 2D CAD-aided design is time-consuming and labour-intensive^31^. Therefore, BIM-based digital design technology is adopted by designers^32^. BIM technology can optimise the design of specific processes in construction, and the optimisation results can be visualised through its visualisation feature. Additionally, it can improve the coordination of building design and avoid later modifications^33^, promoting the integrated application throughout the life cycle and strengthening the digital collaboration of design, production and construction^32,34^. The most distinctive feature of BIM is parametric design, which transforms design concepts into parametric models, buildable shapes and components, etc.^35,36^. Parametric design methods mainly include object-oriented programming, visual programming and functional programming^37^. Among them, the object-oriented programming method is adopted in this paper. Parametric design can harmonise design patterns, improve design efficiency and optimize integrated design^33,38^. The current BIM-based platform is supported by parametric design aids such as Planbar, BeePC and PKPM-PC. Furthermore, PC components are generated by free modelling or by splitting the overall model. The rebar lengths, buried component locations and connection nodes of the BIM model have been modified. However, the design process is still limited by long lead time, large component differentiation, high design effort, and disconnection between design and production.

To adapt to the industrialized design and production of prefabricated buildings, studies have been conducted to deal with a large number of PC components, heavy repetitive work and low standardization in prefabricated buildings to standardise the PC component design and production. Based on BIM technology, Chen et al.^39^ carried out parametric research of components and formulated standards for prefabricated building disassembly and PC component modelling to improve design quality and speed. Nath et al.^40^ integrated information from design to production from three dimensions: product, organization, and process, which shortened production time and increased overall productivity. Isaac et al.^41^ proposed a graph-based approach to explore the optimal modularity of PC components. Bai et al.^42^ constructed a generic library of prefabricated structural components using graphical media maps and combined digital graphical information from BIM with engineering and production data for a more efficient design process. The above studies, although not introducing DFMA, facilitated the industrialization of the design and production of PC components. However, they failed to consider production and process information, resulting in a poor connection between design and production.

In this paper, we propose a DFMA-oriented modular design method for vertical PC components of assembled buildings. The modular hierarchy resulting from secondary splitting was not yet involved in the object of current PC component design research. The secondary splitting of PC components into a variety of standardised modules is applied. This paper aims to propose a design method that can solve the diversity of designs while standardising the components. It will enable all components to be designed quickly and accurately in a standardised way. In addition, it can increase the utilisation rate of moulds and production efficiency and standardise the design, production and installation.

## Secondary splitting modularity

**Figure 1:**
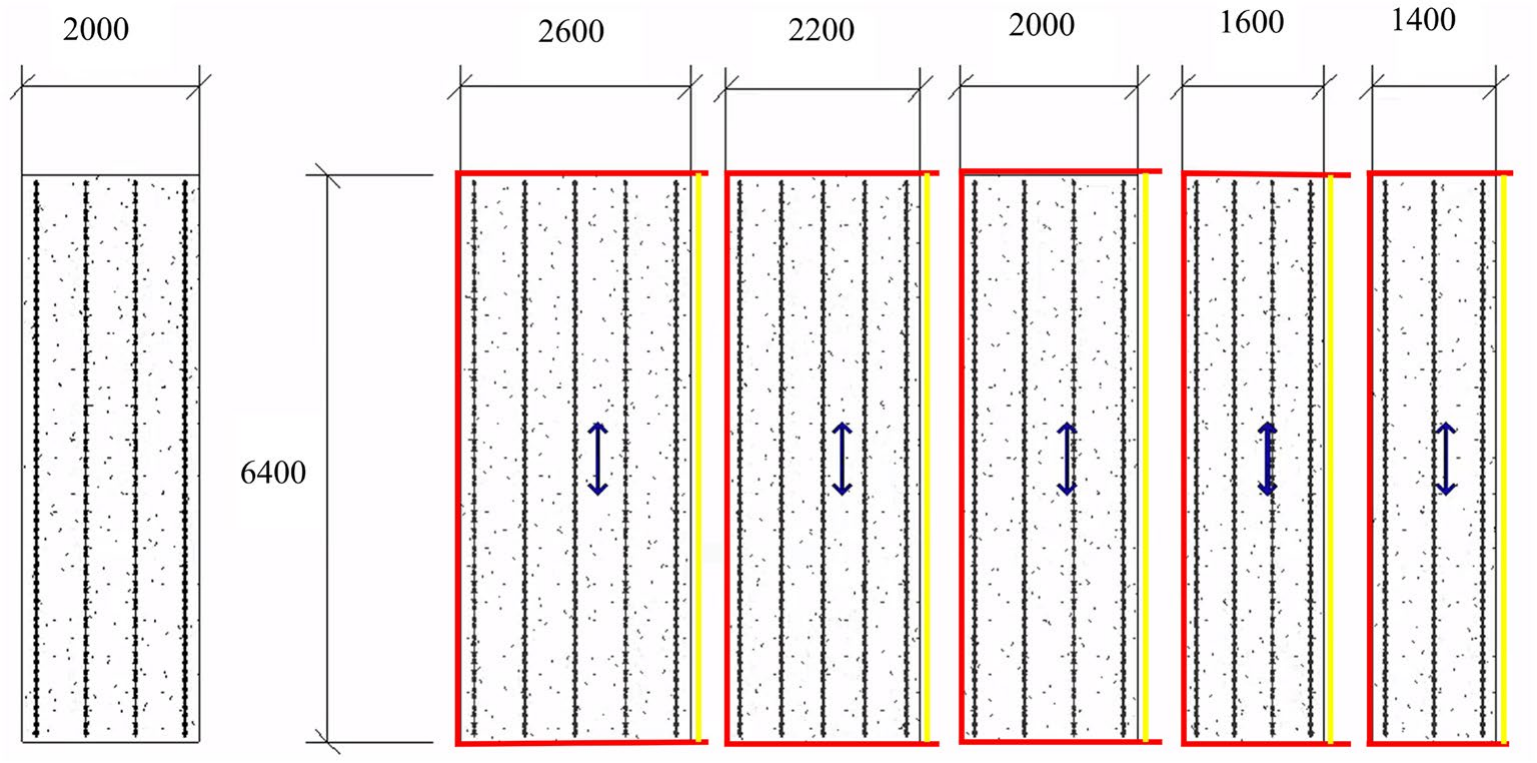
Standardised mode of prefabricated building laminates.

The spatial layout and function of the assembled building can be divided into horizontal and vertical elements. To standardise the design of vertical PC components is to standardise the design of them as products. There are two models for standardising component design, namely, the top-down split model and the bottom-up combination model^35^. The top-down splitting model can be used to perform personalised solution design. In the design process, it is split into standard and non-standard components. Standard components can be determined by maximum convention as the highest repetition rate or by unifying the length of the two sides according to a modular design. Figure 1 shows the standardisation of the laminated panel element design using a top-down model. The fixed edge touch is in red and the removable edge moulding is in yellow. The bottom-up combination model can produce standardised components, parts and modular spaces that will be combined to form diverse buildings based on the productisation concept. In this paper, a new modular design approach for vertical PC components is explored using a bottom-up combination model for standardization.

**Figure 2:**
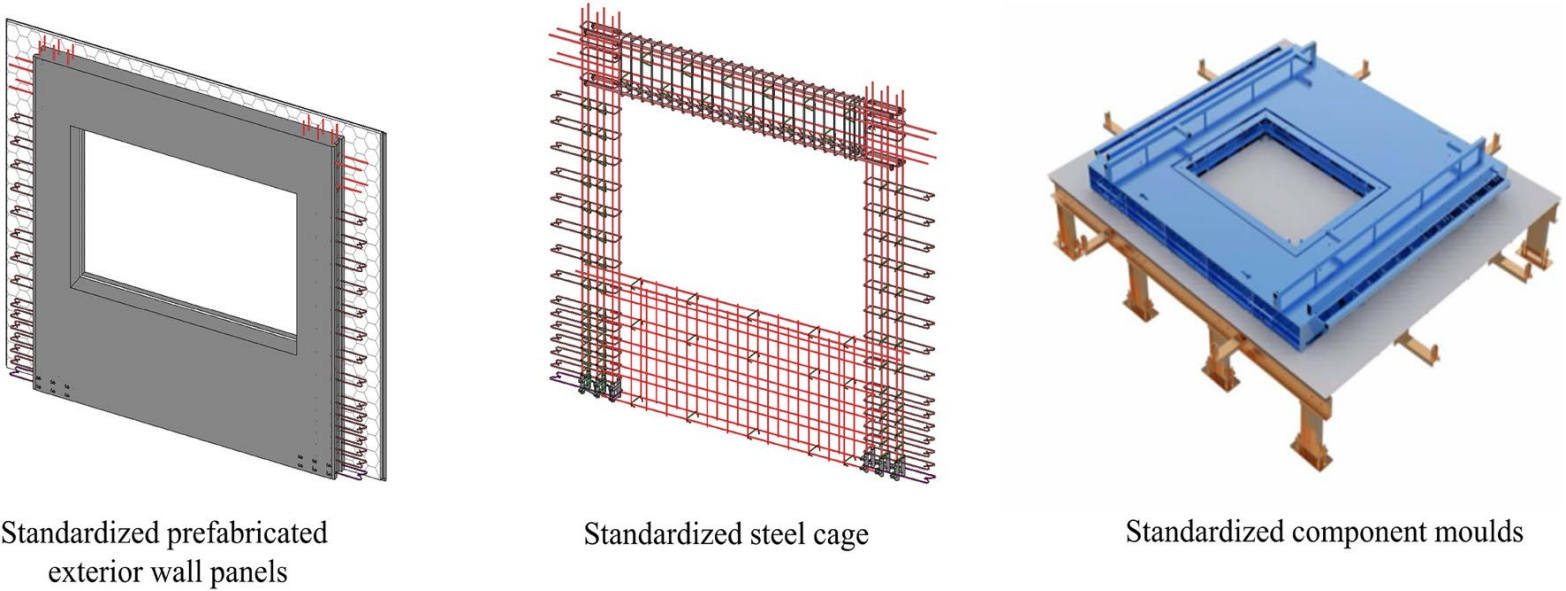
Integrated standardised design technology of PC components, steel cages and moulds.

This new design approach is aimed at vertical PC components. The hierarchy of standardised objects for vertical PC components is established based on the component level and the element level. The main standardisation methods include the typification and specification of vertical PC components. Typification refers to the classification of components into laminated panels, external wall panels, internal wall panels, staircases, etc. Specification is the standardisation of the dimensions of each element type. There are fewer specifications and more combinations^43^. The objects at the element level are mainly reinforcement cages and formwork. The main standardisation methods are the standardisation, unitisation and modularisation of reinforcement cages and the standardisation of formwork. The standardisation of the design of the elements helps to save design time and costs and to improve the quality of the design. The goal is to standardise the design of elements, cages and moulds in component design. As shown in Fig. 2, the PC components, the reinforcement cages in the components and the moulds required for factory production are all standardised. In this design method, it is necessary to standardise the component level and element level, and it can also standardise the diverse PC components split from various prefabricated buildings.

**Figure 3:**
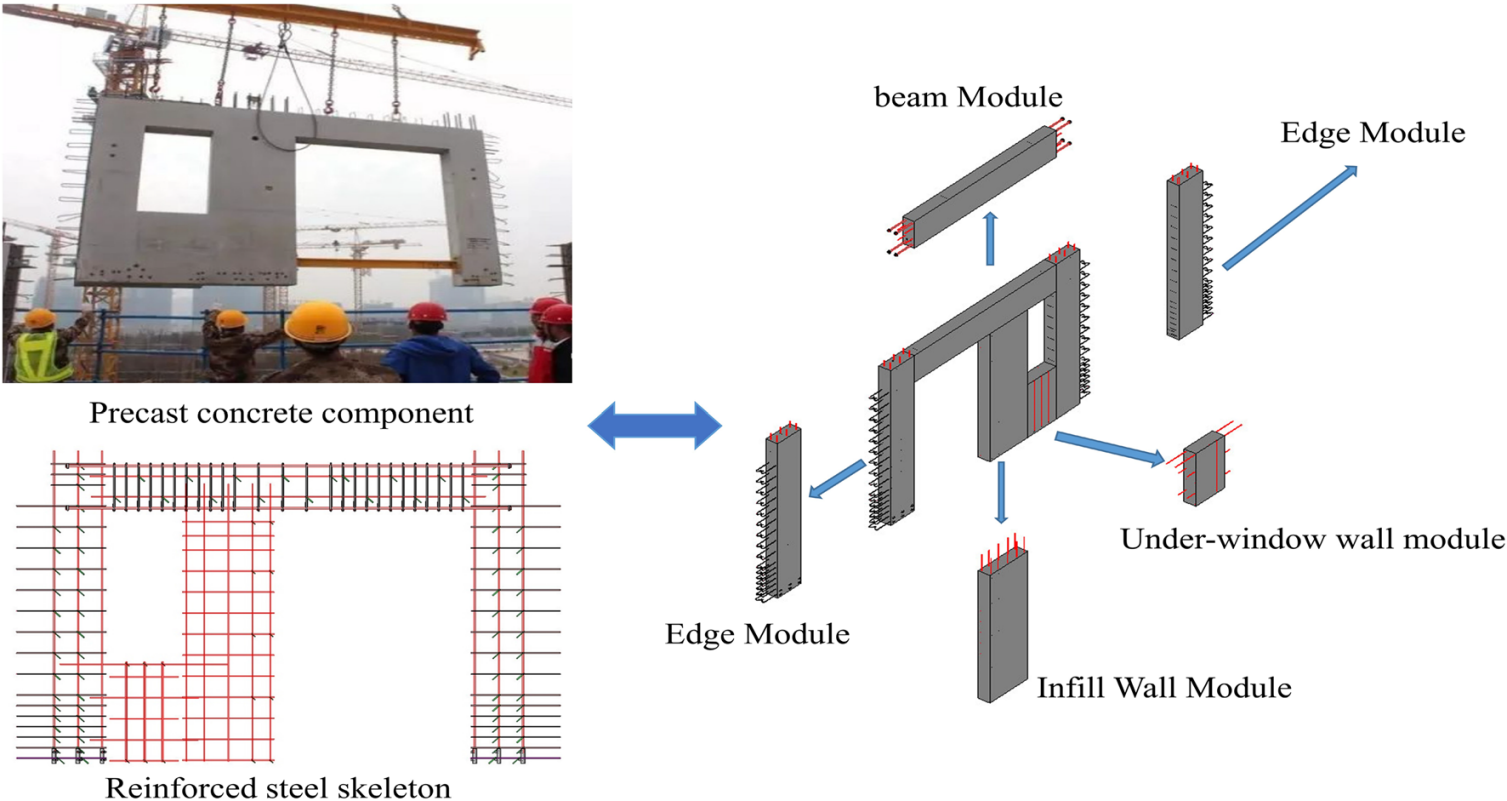
The secondary splitting modularity of the PC component.

This paper takes inspiration from standardised design in the study on standardisation in the design and production of PC components. The use of both specification and modularity for components is explored. A module level is added between the vertical PC component “element level” and the “component level”, and the contradiction between design methods and production methods is regulated through intermediate-scale modules. At the same time, parametric design methods are used to improve design efficiency. The components are split into various standardised modules according to their characteristics. The standardisation of PC components is achieved through the standardisation of secondary split modules. When splitting PC components, the front-end design, factory production and on-site construction should be first considered. Secondly, the standard is based on a summary and analysis of the accumulated production experience of a large number of assembled buildings. This study emphasizes modular splitting at the “component level”. At the “element level”, the rules of the disassembled modules are combined into a standard form, for example, the rules for the layout of reinforcement in PC elements are standardised, which is also in line with the modular and standardised nature of DFMA. Figure 3 illustrates the secondary splitting modularity of the PC component. The vertical PC component with three window openings is split into standardised modules such as the beam module, the left (right) edge module, the infill wall module and the under-window wall module. The modular design approach is fully reflected. The standardisation of the assembled PC elements is achieved by the standardisation of the secondary split module combination.

There is no consistent definition of modularity in the construction literature. In many cases, modularity is used as a volumetric unit (e.g., “boxes” that are prefabricated in a factory and then transported to the site). Modules usually refer to products that can be produced off-site, such as transportable units and components^44^. In this paper, the module applied to PC components is defined in accordance with production standards, structural stability and other commonalities of secondary split modules. The beam module, for example, conforms to the commonality and combinability of the components and improves production efficiency.

## Modular design

### Coding of modules

The module and model information from the database is retrieved by compiling a structural code for the module. The module's code consists of key parameters that determine its characteristics. For example, edge modules are influenced by factors such as floor height, seismic rating and concrete strength and all of these elements are present in the code. Accessing modules by coding is also unique and logical^45^. An example of coding for the edge member modules is shown in Table 1. The strength of PC is in the range of C30 to C60; seismic rating is from class 1 to class 4; the width of the module is 200 to 600; the sleeve size is 12 to 18; the storey height is 2900 to 3100.

**Table 1:** Example of edge module code.

Concrete strength	Seismic rating	Width/mm	Sleeve size	Floor height/mm	Examples
C30	E1	200	12	2900	E1C30H2900L200C12T4ZK
C35	E2	350	14	2950	E2C35H2950L200C12T4ZK
C40	E3	300	16	3000	E1C30H3000L200C12T4ZK
C45	E4	350	18	3100	E1C30H3100L200C12T4ZK

### Linkage of modules to structures

Through previous research on projects, it has been found that in a single member, the reinforcement in different zones will take on different roles, and the reinforcement playing the same role will essentially be of the same size. The elements are divided into standard functional modules. The left and right supports are classified as left and right edge element modules, which act as vertical forces on both sides of the element; the remaining parts are infill wall modules, beam modules, etc.

The PC components are split into the component level and element level. For the element-level objects used for structural safety, standardised reinforcement cages are adopted to improve standardisation. The reinforcement cage for the modules ensures the standardization of the design and manufacture of PC elements and structural safety during design. Firstly, when the modules are assembled into PC elements, through the secondary development of BIM software, the non-stressed module reinforcement is threaded through to achieve the overall structural stress of PC elements. Secondly, during module generation, the designer controls the diameter of the reinforcement bars in the cage by entering the structural parameters of the module. For example, the cross-sectional area of the reinforcement is entered to control the diameter of the reinforcement, thus ensuring the safety and stability of the PC component at different loads. The dimensions of the reinforcement bars in the cage are derived from extensive empirical analysis and incorporated into the design rules of the module.

**Figure 4:**
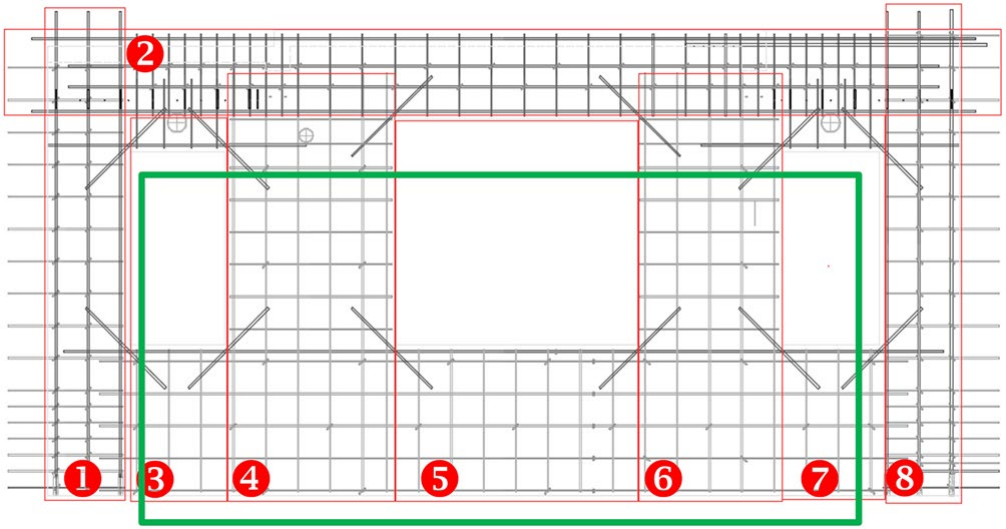
Module composition in PC components.

Figure 4 shows the vertical PC component with the reassembled modules, including the left edge module ①, the right edge module ⑧, the under-window wall modules ③⑤⑦, the infill wall modules ④⑥, and the beam module ②. In the green box in the diagram, modules ③–⑦ are assembled into the unstressed part, and the stressed part is assembled by the left (right) edge modules on both sides. Then, the design process is simplified by a modular, parametric design approach. After receiving the complete design drawings and bill of quantities of the PC components output at the design stage, the factory does not manufacture PC components according to the module hierarchy, but rather the complete PC component design drawings. Finally, to ensure the stability and safety of the structure, the reinforcement cage can be adapted to the design rules of the module according to the codes and standards of different regions, for example, the design parameters of the spacing, diameter and anchorage length of the reinforcement bars, to ensure the universality of this method.

### Linkage of modules to manufacturing

**Figure 5:**
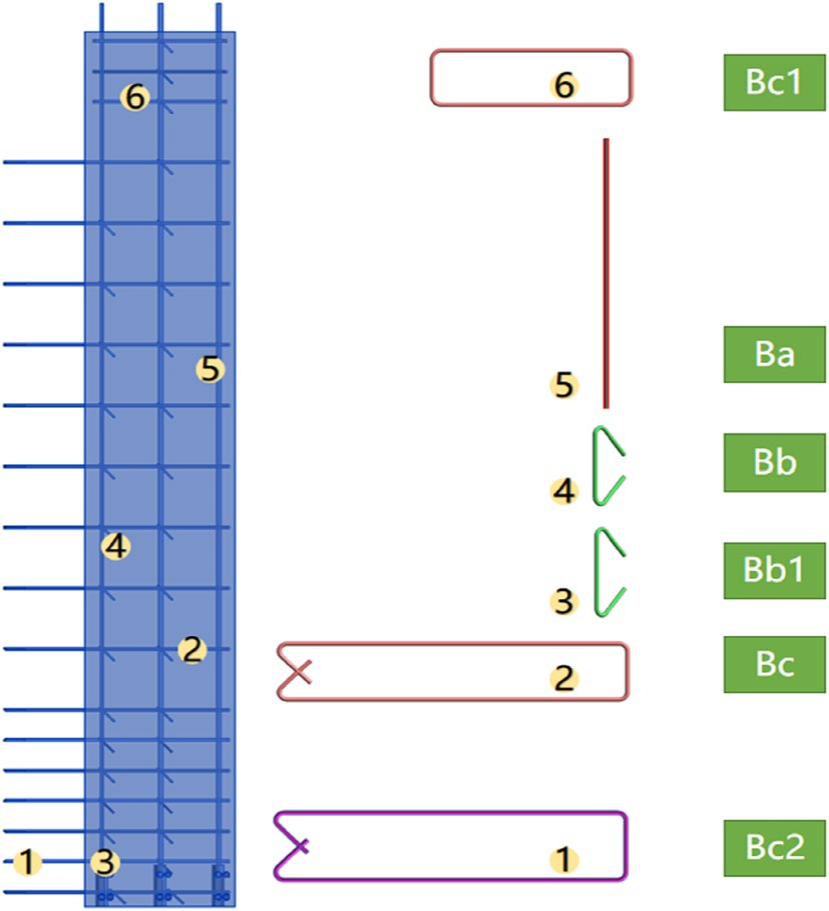
Information on the production of left-edge components.

The components can be disassembled into design, production and material information. The reinforcement bars in the modules have a fixed number and a corresponding processing table. The design and material information can be exported on the premise of a clear module. The corresponding rebar processing information can be generated provided that the corresponding numbers are available. The modules also have corresponding folders and graphic files so that the corresponding production information can be entered during the design phase to link the modules to production. When the module has associated production information and it is called up as part of a component, this production information can be generated and output simultaneously. The number of the left-hand edge component and the corresponding machining information are shown in Fig. 5. Modules can be disassembled into different material information such as reinforcement and attached prefabricated parts to match the manufacturing information required by the factory in manufacturing the components.

### Linkage of modules to DFMA

This paper introduces DFMA, where “manufacture” refers to the manufacture of individual modules and “assembly” refers to the combination of modules to form components. DFA is used in the PC component generation process when the structural design is proven to be feasible. When materials are updated or processes are upgraded, a better design concept can be developed and the module generation graphics can be updated. DFM is the detailed design for minimum manufacturing costs, including the design of vertical and horizontal reinforcement locations, the design of reinforcement mesh and cages, and the design of the form and location of tension bars. Once the DFM is verified to be feasible, the generated model of the module can be updated.

**Figure 6:**
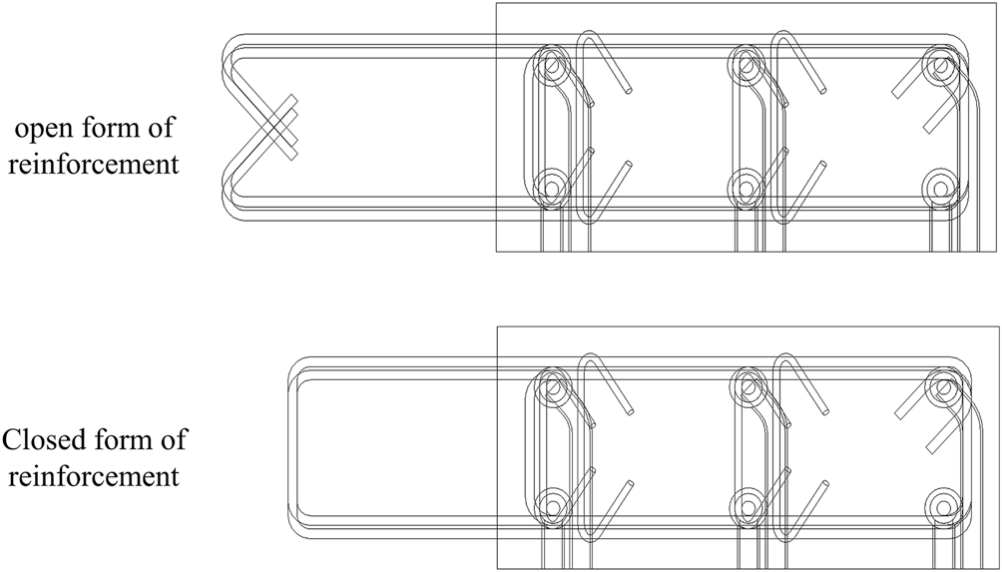
Reinforcement forms of edge modules.

Module design rules are not fixed. When information on modules with a more generalised structure and lower production costs is available, new production model information can be entered for iterative upgrading of the module. For example, in edge modules, the location of vertical bars and horizontal hoop exits of edge members are designed so that they do not collide. The use of reinforcement in the form of steel bars, as shown in Fig. 6, makes this form easier to work with. From a production point of view, the short length of the reinforcement reduces production costs and offers versatility. Therefore, iterations of the generated model are completed using the closed-ended reinforcement form of the edge member. At the same time, the design takes into account the need for assembly, which also allows for better assembly of the module.

## DFMA-oriented parametric design flow

**Figure 7:**
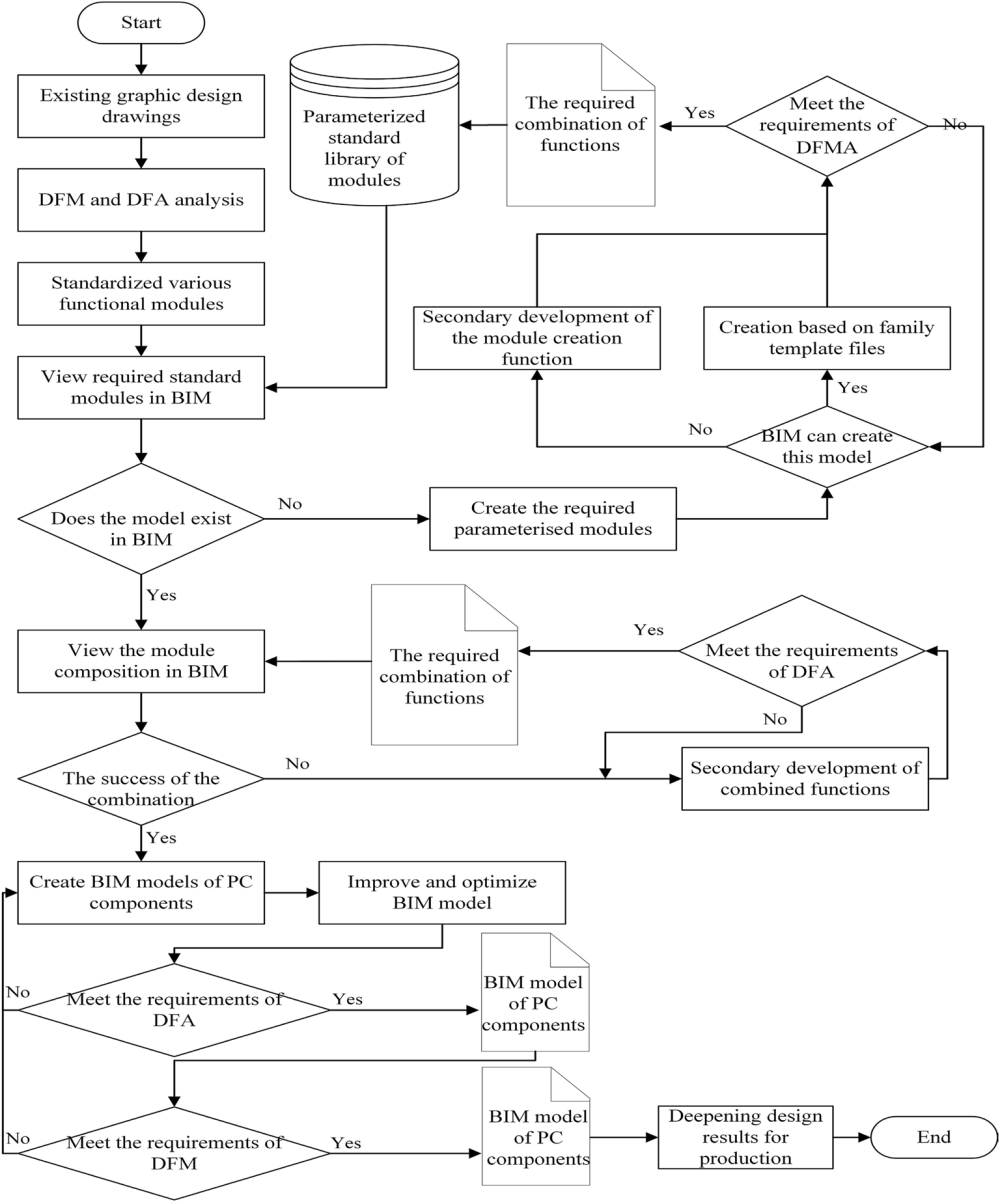
DFMA-based parametric design flow.

In the PC component design process, BIM is the key technology supporting DFMA, and DFMA makes BIM more suitable for PC components. The DFMA-oriented parametric design concept is based on the organic combination of BIM, DFMA and parametric design. Figure 7 shows the process of introducing DFMA into the parametric design of vertical PC components, which is mainly divided into the following steps. Firstly, data are analyzed. A large number of PC component floor plans have been retained from assembled building projects that have been built or are under construction. Design analysis is carried out for the plans, especially DFM and DFA analysis. The drawings are analyzed for item information, reinforcement information, spatial layout, and pre-buried component locations of the components. Secondly, PC components are disassembled. The composition pattern of vertical PC components is studied, and the modular categories of the constituent components are established. The PC components are split into various standardised modules and the design drawings of the modules are obtained. The design drawings are analyzed to determine how the PC components should be split and to meet the technical requirements and cost requirements of manufacturing and assembly. Thirdly, standard modules are created. The rules for the composition of the modules, the laying of reinforcement, and the drawing of modules are studied and developed. Fourth, parametric modules are created. BIM software is checked to determine whether there is a BIM graphic of the module. If BIM does not have the corresponding parametric standard module and module reorganisation into PC components, the module needs to be created through the secondary development of the BIM software or through the family template. After successful creation, whether it conforms to DFMA requirements is checked. The created BIM modules will be saved in the corresponding module library and the subsequent design of the PC components will be completed by calling the corresponding modules. Finally, Application of Parametric Module. The combined BIM components will be improved and optimised by adding appropriate accessories and insulation panels. At the same time, the perfected BIM model of the PC component is checked for DFA and DFM. Once certain manufacturing and assembly requirements are satisfied, the subsequent design process is carried out and the factory floor plans for production are output.

## Assisted DFMA-based parametric design approach

### Module development process for DFMA and BIM

**Figure 8:**
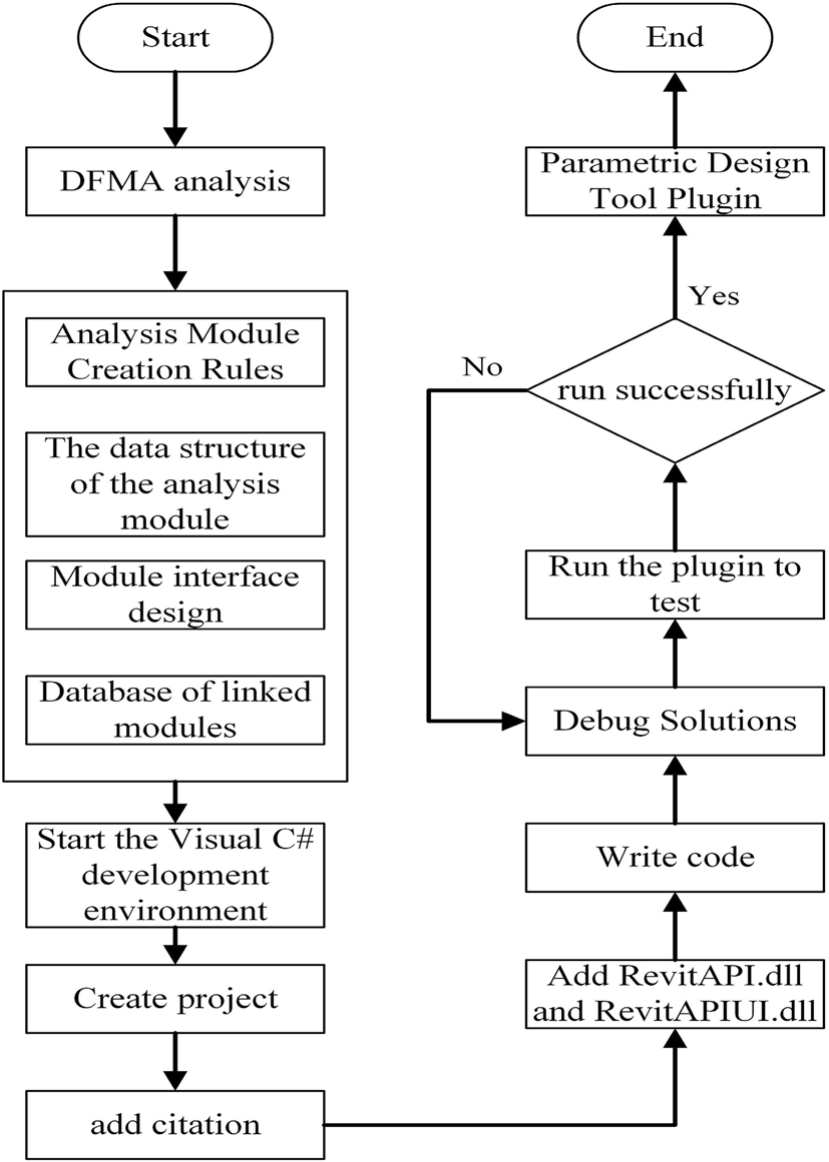
Module development process for DFMA and BIM.

In the DFMA-oriented parametric design process, the BIM software is used based on the implementation of the parametric design of the modules. As the diversity of modules increases, the BIM software cannot support the creation process of all modules. Usually, BIM software does not contain parametric components at the module level. Parametric components can only be created in certain ways. The first way is the creation of family-based prototype files. The second way is the secondary development of the BIM software to create the BIM model of the module. In this paper, the parametric BIM modules are created through secondary development tool software. The development platform is Revit2016, the BIM-based modelling software. The programming software is VS2015 (Microsoft Visual Studio 2015) and the object-oriented programming language is C#. Revit2016, developed by Autodesk, is currently the mainstream BIM modelling software and one of the most widely used software in the BIM system for the construction industry. Its biggest advantage lies in parametric design^46^. The Revit API can be used to create parametric add-ins^47^. A parametric software interface is developed on the basis of Revit, as shown in Fig. 8. Firstly, manufacturing and assembly-oriented component analysis is performed to understand the development goals and details that are more suitable for modularisation. Secondly, factors such as module creation rules, combination rules, data structure and interface UI are analyzed. Thirdly, the appropriate development language and development platform are selected for the development of tool plug-ins. Fourth, module rules, databases, etc., are built into the tool software by writing codes. Finally, modifications and debugging are performed to complete the development of the parametric tool software.

### Parametric module creation based on family templates and DFMA

A tool based on the secondary development of the BIM software, which allows the parameterisation of modules, is developed. When creating a module, the model is created by calling the family template documentation environment named “ModuleTemplate.rfa”. The template has a built-in family containing the components, which can be saved and quickly used to create the next module, increasing the speed of modelling. The parametric design process of the modules is divided into two parts. Firstly, a BIM-based parametric design tool software is created through secondary development. Secondly, the components that make up the module, such as reinforcement, section dimensions and the various elements of the module, are parametric elements that can be adjusted by changing the parameters. Finally, the modules made up of components can be adjusted in the BIM environment according to design requirements to realize a parametric design process.

**Figure 9:**
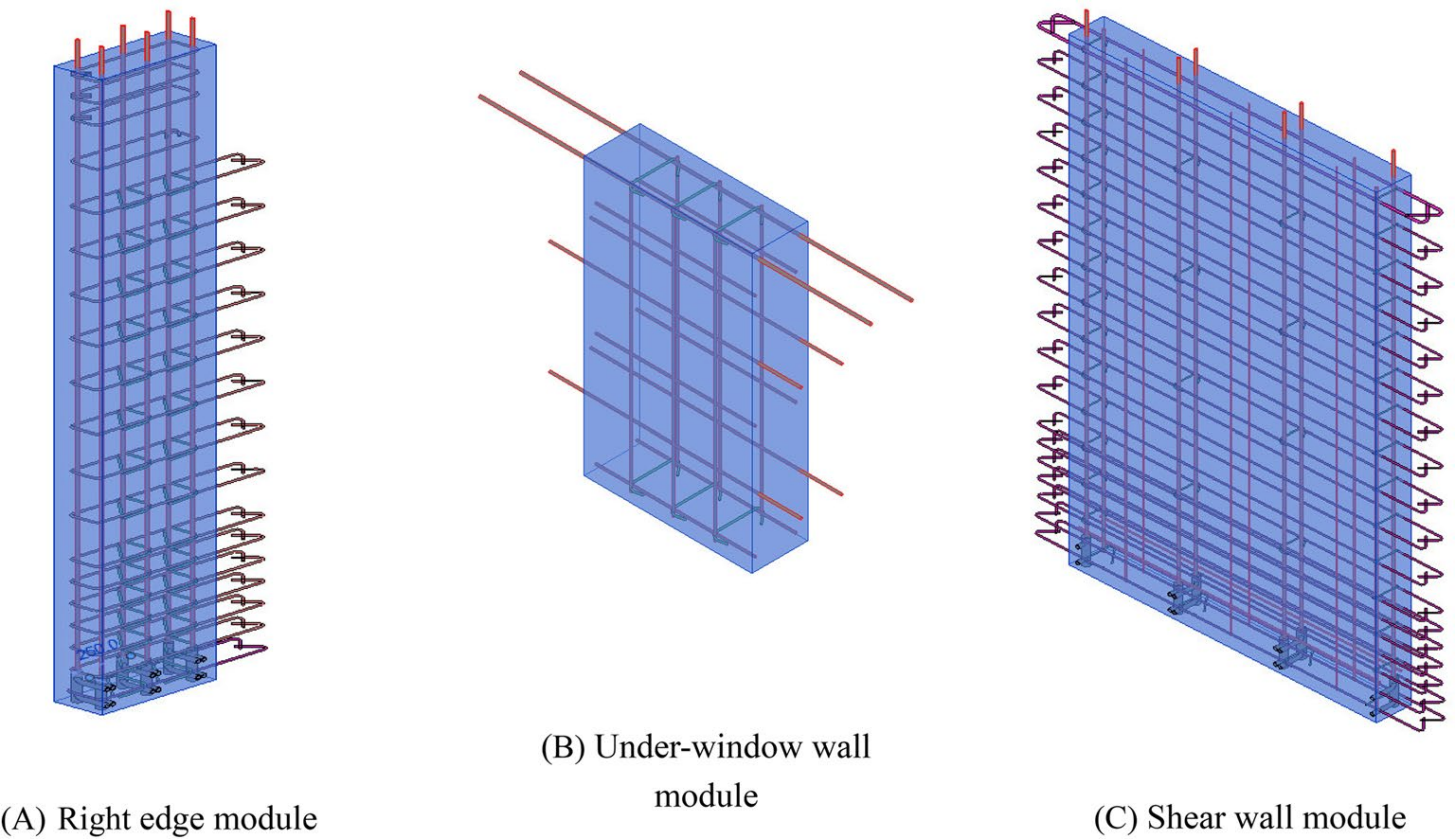
BIM model of modules.

The creation of modules is linked to the PC component information. The BIM model of the module is built by entering the corresponding parameter information. It is necessary to verify that the module meets the design and production requirements to ensure that it is manufacturable and assembleable. Adjustments are made if modifications are required. Additionally, unique codes related to the structural parameters are created for matching and retrieving the modules. Then, the module BIM model, codes and other design information are saved to the module database to form a parametric module library. Figure 9 shows the parametric BIM model of the modules in Revit, including the right edge module, infill wall and under-window wall modules. These modules are parametric and can parametrically adjust the components in the module according to the design and manufacturing requirements.

## Assembly of modules

### Assembly of regular PC components

**Figure 10:**
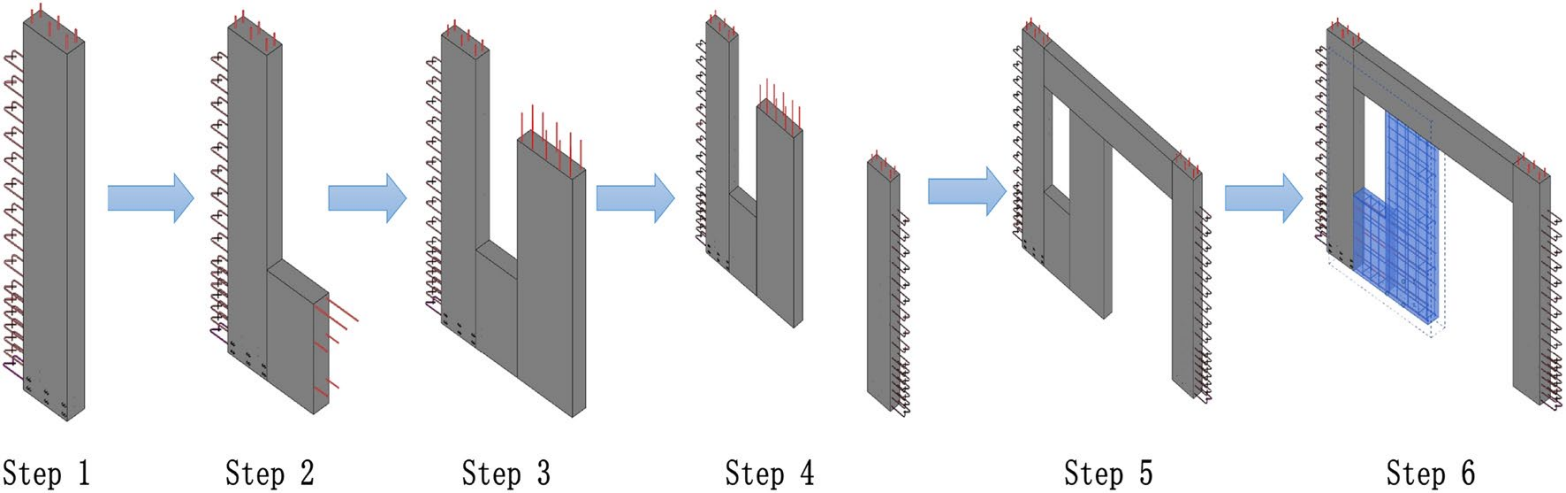
BIM-based parametric module combination process.

Parametric modules created based on BIM software are saved in a cloud-based module repository for easy upload and recall of modules. The module code is generated by entering the structural information of the module into the module database. Based on the codes, multiple modules are retrieved to form components of various shapes and sizes. The developed assembling algorithm can be used to assemble a wide range of components, ensuring that the modules are assemblable and meet the requirements of DFMA-oriented design and production. For conventional vertical PC elements, the assembly process is shown in Fig. 10. Depending on the different design requirements, the developed assembly algorithm is used to call edge modules and infill wall modules to form PC components. After the combination, the reinforcement in the non-stressed modules is penetrated, as shown in Fig. 10(Step 6).

### Assembly of non-regular PC components

**Figure 11:**
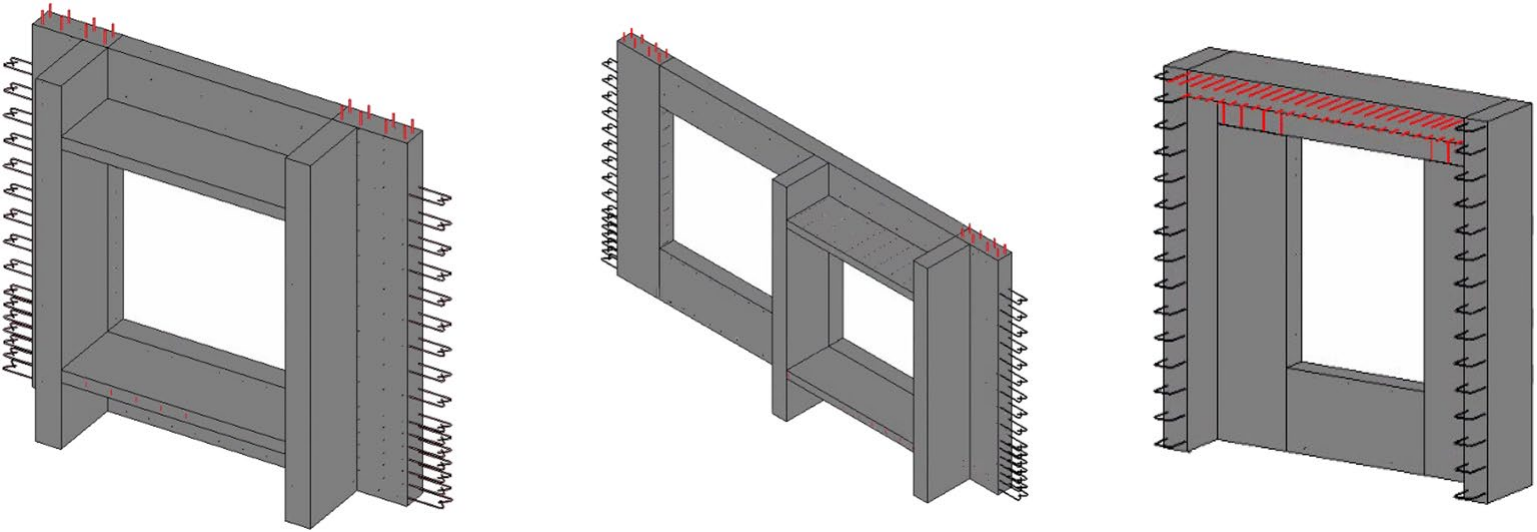
Non-regular PC components—Floating windows.

Assembled buildings can be split into the conventional vertical PC elements commonly used on construction sites. However, the increase in people’s aesthetic and functional needs for buildings has led to a diversity of designs and many unconventional PC elements. Floating windows, for example, have a variety of shapes and complex types that are difficult to model quickly in the design process. In this paper, a modular design approach is introduced, allowing rapid modelling of unconventional PC components by retrieving the corresponding functional modules. The 3D model of the floating window assembled by the module is shown in Fig. 11, confirming the universality of the design method in this paper, which can facilitate fast modelling and speed up the design.

### Complete PC components

**Figure 12:**
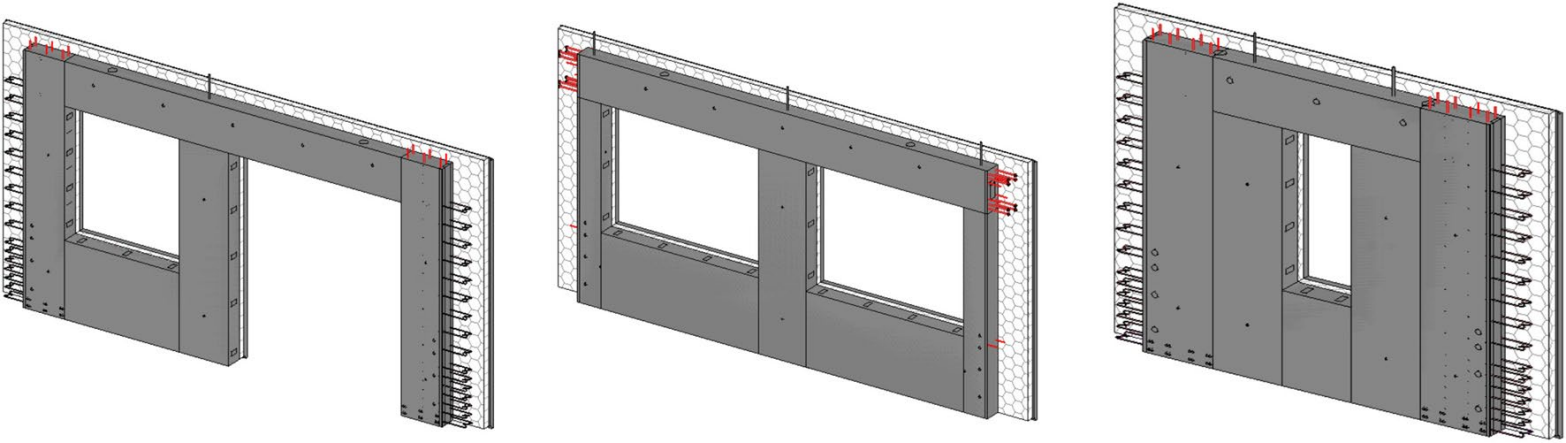
A complete parametric PC component is formed.

The PC components are combined, with the corresponding accessory parts added. Depending on the design requirements, these accessories include insulation and prefabricated parts. The insulation layer is used as an integral part of the PC component for the decorative insulation of the structure. There are various pre-buried parts, mainly including production pre-buried, construction pre-buried and electromechanical pipeline pre-buried parts. Different pre-buried parts are in different locations, which makes the integration and standardisation of the PC component design more difficult. The standardisation of pre-buried parts is improved to make the pre-buried parts common or universal, and the standardisation of components is also improved. Except for the pre-buried parts that need to be added manually, some pre-buried parts are added automatically in the program with one click. The rest of the embedded elements can also be arranged according to the design scheme with a single click. Finally, a complete parametric PC component is formed, as shown in Fig. 12. Revit can generate plans, elevations and 3D drawings simultaneously during the design process. The traditional design process lacks thinking about the production process. In this paper, for the design results of production, BIM software can be used to create design drawings and quantity lists for production and processing.

## DFMA-oriented whole-life optimization process for assembled buildings

**Figure 13:**
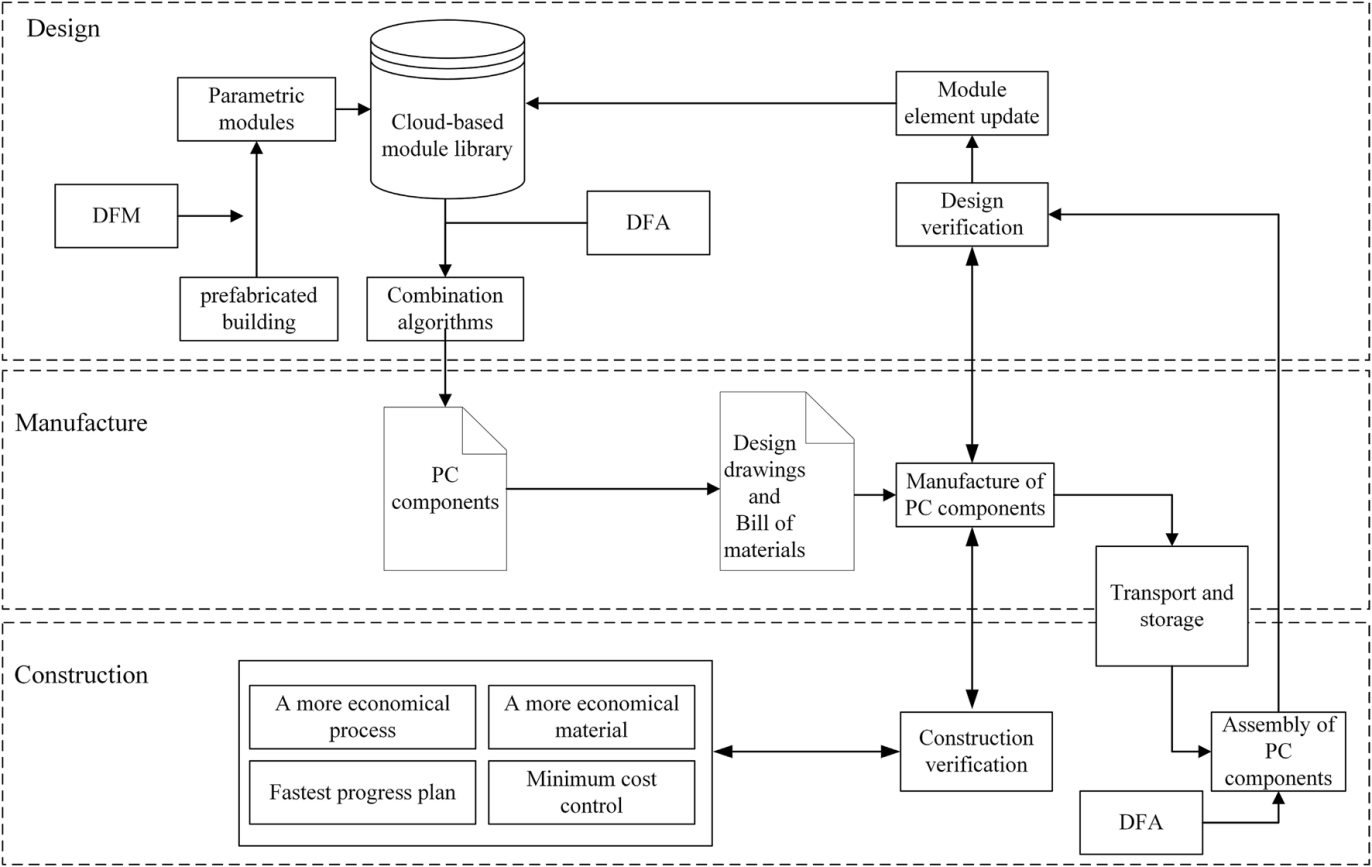
DFMA-oriented whole-life optimization process for assembled buildings.

During the standardisation and digitisation of PC components, BIM is the basis for digital and intelligent PC components. The upgrading of production equipment and production methods provides the basis for the standardisation of production processes. The modular and parametric design approach in this paper provides a reference for the digitisation of the design, production and installation of assembled buildings. This paper also explores a whole-process design based on the cloud-based module library. The cloud-based module library enables the integration and digitisation of the whole process of assembly design, construction and installation and is used as the basis for a data-driven whole-process design process, as shown in Fig. 13. The whole process flow, with modules formed by the secondary splitting of PC components, runs through the entire process of design, production and construction. It also facilitates the design, production and construction projects in engineering, procurement and construction (EPC) that can be used to maximum benefit.

In the design phase, after corresponding modelling and structural calculations, the assembled building can be split into individual PC components, and the corresponding PC component split drawings are formed. The parametric module is created by entering the structural parameters of the module through the developed parametric design tool, and it meets the requirements of DFMA. Firstly, DFM is mainly applied to the secondary splitting of the PC components of the assembled building, where the modular design is introduced. Secondly, design rules for split modules are solidified according to the needs of manufacturing and assembly. Additionally, DFA is mainly used to simplify the structure of the module and reduce the number of modules to facilitate assembly. The split modules are designed with the consideration of the requirements in manufacturing and assembly. Parametric design methods are introduced, and corresponding design tools are developed to optimise design speed. The parametric modules are saved in a cloud-based library of parametric modules. For different PC components, multiple modules are retrieved according to codes to form components of various shapes and sizes. The assembly of PC components with fewer specifications and more combinations is achieved, which conforms to the diversity of designs. The developed assembling algorithm is capable of assembling a wide range of components, adapting to the actual needs of most projects and ensuring the assemblability of the modules.

In the manufacturing phase, the design and production of PC components are in line with DFMA requirements. Revit can generate floor plans, elevations and 3D drawings simultaneously during the design. The traditional design process lacks consideration of the production process. Therefore, for the design results of production, BIM software is used to create design drawings and engineering quantity tables for production and processing in this paper. After manufacturing in the factory, the finished PC components are transported to the assembly site or yard for subsequent construction needs.

The design of assembled buildings is linked to the standardisation of production. To standardise the design process, after the preliminary structural design of the assembled building, the component split design is carried out, followed by the component design. The factory obtains the design drawings and list of quantities for the production and processing of PC components, which are finally transported to the construction site for assembly^48,49^. The designs of different assembled buildings vary, which requires that the PC components, production moulds and production materials for different projects are almost always customised for the project. The difficulty in reusing greatly impacts the overall schedule and costs^50,51^. The production process of PC components is a value-added manufacturing process from material, processing, assembly to transport^52^. The current design goes directly from machining to assembly without considering the production process, and the design drawings impede the component production process, which sometimes greatly reduces production efficiency^14,53^. To standardise the production of PC components, the mould should be adjusted to a flexible size to increase its suitability.

**Figure 14:**
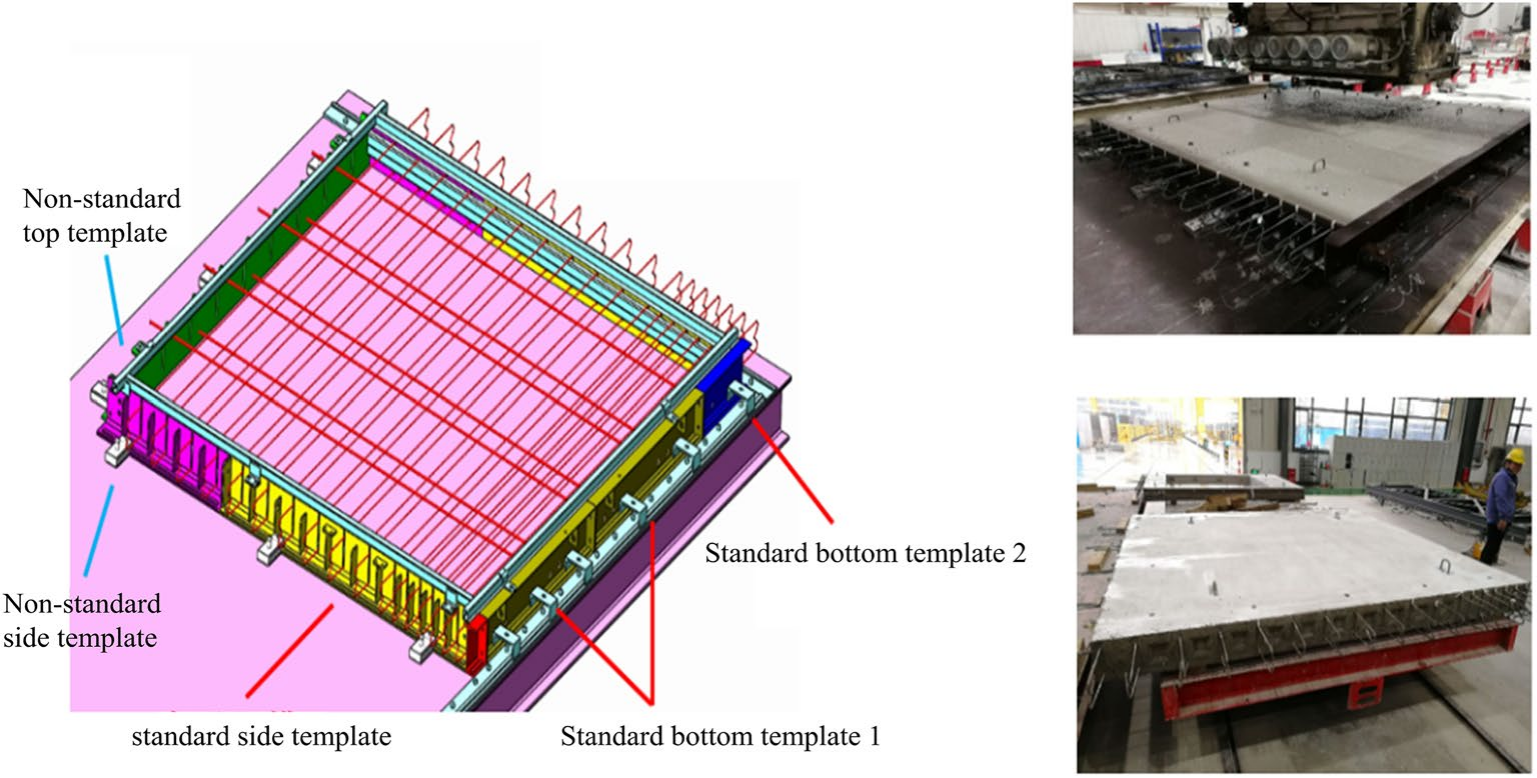
Mould combination diagram.

Therefore, through the parametric design method of DFMA in this paper, the needs of component production are fully taken into account during design. This design approach simplifies the form of the components during design and facilitates manufacture. It is combined with flexible manufacturing methods that allow for standardised production by using a common mould to produce components with unique characteristics. A partially fixed, combination mould with few specifications and many combinations is developed, as shown in Fig. 14. The combination moulds consist of standard and non-standard bases, side moulds and top moulds. During the production, the moulds can be combined to form the required ones matching the sizes of PC components. The combination enables the production of a diverse range of PC components with good manufacturability, reduces the production costs of the moulds and increases the reusability of the tooling.

In the assembly phase, DFMA enables good assembly of the modules designed in the design phase. In this paper, assembly is manifested in two ways: the first is the reorganisation of the secondary split modules into PC components through a developed combination algorithm, which occurs in the efficient assembly of the modules; the second is the process of assembling PC components into an assembled building during on-site construction. The best PC components with good manufacturability and assembly are transported to the construction site for installation. The process of installing PC components on site is also a practical validation of the design and production process. For example, whether the secondary split modules can meet factors such as the minimum cost plan and the fastest schedule during construction. Moreover, the process can also be fed back into the component design and production process for optimisation and adjustment. After validation, the parametric modules that do not conform to the concept of DFMA are updated with module elements and the module library. The whole design process meets the requirements of DFMA for manufacturing and assembly and facilitates the entire process of design, production and construction of modular assembly buildings. It enhances the coordination of the whole process, reducing costs and facilitating production.

## Results and discussion

The design methodology proposed in this paper was applied to 38 assembled building projects built or under construction by a construction company in Wuhan, China. The 38 projects have a total of 1806 types of vertical PC components. This design method can complete the design process for 1766 PC components (96.2%). Modules that need to be expanded account for 2.1% and components that need to be communicated and adjusted with the structural design account for 1.7%. It is proven that the modular design approach can standardise the design of the majority of vertical PC elements. This is a very mature design method for assembled PC components, as it can provide design drawing files and processing data information that meet the project requirements.

Building 9 in a project was selected to verify the changes in the design approach proposed in this paper and the common design-aid software approach on the project. The project has 2 underground floors and 33 above-ground floors, with a total residential construction area of approximately 11,433 square metres. The pre-fabricated assembly rate of the individual blocks is 46%. PC components are mainly used for standard floors. The vertical PC components are studied, with external wall panels numbered YWQ1–YWQ21 and internal wall panels numbered NQ1–NQ18. The vertical PC elements of the project were modelled in a refined manner. The average time spent on modelling to drawing with the two deepening methods in a single component was compared under the same deepening criteria. The comparison results are shown in Table 2. It is proven that the DFMA-based PC component design approach is effective in increasing design efficiency by an average of more than four times.

**Table 2:** Speed comparison of design approaches.

Type	Name	Numbers	Ancillary software design method/(min)	DFMA design method/(min)	Time savings/(min)
External wall panels	YWQ1-YWQ21	66	22	4	18
Internal wall panels	NQ1-NQ18	48	15	3	12

Different projects call on modules in the database to deepen the design and the supporting moulds can be called directly. Quantitative analysis of the vertical PC components for building 9 was performed. The number of moulds required for the production of the components was compared to verify the improvement of the standardisation level. The number of moulds used for different components of the project is shown in Table 3. The study proves that the design method and module library in this paper significantly reduce the number of mould sets required for component production, which improves the versatility of the moulds, reduces the production cost of the moulds and improves the standardisation of the production.

**Table 3:** Number of mating moulds.

Type	Name	Number	Ancillary software design method/(min)	DFMA design method/(min)	Moulds saved/(min)
External wall panels	YWQ1-YWQ21	66	66	32	34
Internal wall panels	NQ1-NQ18	48	48	25	23

This paper introduces DFMA and proposes a parametric design method based on DFMA, taking assembled vertical PC components as the research object. By innovative secondary splitting of components into various standardised functional modules, a standard module database is established. The design rules for the modules are formulated and built into the developed BIM tool software. Parametric modules are created and combined into parametric PC components to complete the design. When the parametric PC components are generated, the design and production information is synchronised and the digitalisation of the components is realised. Through the analysis of more than 60,000 design drawings, a variety of functional modules such as beams and edge members are summarised. Different modules, in the form of rectangular combinations, can form more than 80,000 vertical PC components, comprising a PC component database. The approach proposed in this paper can achieve quick and accurate completion of plan design drawings in a standardized way while improving the use efficiency of moulds as well as production efficiency. The effect of multiple products, wide coverage, fast speed and the same standard is achieved. Through the secondary development of BIM software, BIM-based parametric design tool software is developed, significantly improving the design efficiency.

The DFMA design approach in this paper is compared with the common parametric-aided design software for assembly buildings. The auxiliary software can complete the process from modelling to drawing to achieve the digital design of PC components for assembled buildings. However, it cannot quickly create parametric models of diverse, complex and high-volume PC components. The design results require a lot of modification, which wastes time and manpower, greatly affecting the design efficiency. In this paper, we use modules to form components, similar to building blocks, allowing a limited number of modules to form an infinite number of PC components and can adapt to the diversity of front-end products. For assembled buildings, the splitting also stays at the component level. This paper is oriented to the parametric design process of DFMA and analyzes the secondary splitting design and DFMA with assembled vertical PC components as the object. The data for vertical PC components comes from a large number of built assembly projects that have been tested in practice in design and construction, which is a reuse of production experience. This re-used data also facilitates the link between factory production and site assembly from a design perspective. A standardised parametric module library is established through the secondary development of BIM based on family templates. The module library not only realizes the standardisation of PC components but also optimises the whole lifecycle process of the design, production and construction of assembly buildings. Moreover, the potential of BIM is further explored. The output of the DFMA-oriented parametric design is the building and production information models of PC components, instead of a large number of paper drawings, which facilitates the acquisition and transmission of data during design and production. The main differences in software usage are shown in Table 4.

**Table 4:** Evaluation of design approach.

Evaluation indicators	DFMA-oriented design approach	Non-DFMA parameterisation approach
Design approach	DFMA-oriented and based on parametric standard modules	Integral model splitting or free modelling of components
Data sources	Design criteria drawn from several projects	Integration of current local codes and drawing sets
Modelling quality	High level of model detail without unnecessary manual adjustments and high automation	Low refinement of components and manual adjustment required
Databases	Standardised module database for design diversity across projects	Parametric component library for individual projects
Model information	Models with built-in production information and complete data transmission for subsequent production	Graphical design and lack of production information; data transmission prone to deficiencies
Difficulty of operation	Simple, one-click operation of tool software	Complicated operation; easy to repeat modifications
Production and assembly	Designed with production and assembly in mind, in line with the whole-process design	Less component production and assembly during the design

Each assembled building project goes through design, machining, and production adjustment stages. Design efficiency is increased by more than 80% through the secondary development of parametric tools. The same type of module with the identical form of reinforcement can be produced using one set of moulds for different components, which saves time spent on mould processing and production commissioning. The DFMA-oriented parametric approach can optimise the design process, improve PC component standardisation and reduce costs. It can help to save more than 80% component design costs, reduce component tooling costs by approximately 75% and achieve minimal manufacturing costs.

In this paper, we innovatively propose a modular and parametric design method for the secondary splitting of PC components. Based on the parametric standard module, the module design rules are formulated to realize the data correlation of design and production. The design of the components is standardised, the design and production process is simplified, the production and installation process is standardised, the design cycle is shortened, the production costs are reduced and the requirements of DFM and DFA are met. The PC components produced have good manufacturability and assemblability. This method solves the problem in design processes that rely on personal experience and automates the knowledge of PC component design. This method also reduces the difficulty of promoting assembled buildings and enables the assembled projects to be adapted to different design institutes and different owners. Additionally, the production of PC components is standardised, and the difficulty of access and promotion is reduced, expanding the application scope of assembled buildings.

To realize the DFMA-oriented parametric design, this paper investigates the vertical PC components of an assembled building. A digital-aided design method is established, including the secondary development process of parametric modules for DFMA and BIM and the basic process of creating, combining and applying parametric modules based on family templates for DFMA. The secondary development process of DFMA and BIM solves the functional problems of automated creation and module-to-module connection in BIM software. The basic process of creating, combining and applying parametric modules based on family templates and DFMA solves the problem that split modules cannot be created quickly in existing BIM software due to the lack of relevant parametric components. The design approach in this paper demonstrates the modularity and parametrization of secondary splitting of PC components and the creation of a corresponding library of parametric standard modules using the developed tool software. During design, production and construction based on the parametric standard module library, the modules in the library are continuously optimised according to real data feedback. The optimization effect on the whole life cycle of assembled buildings can facilitate the evolution of PC components to be more easily easier manufactured, more material-efficient and more environmentally friendly.

The design approach in this paper also has some limitations. Firstly, the object of this paper is the vertical PC components of assembled buildings. For horizontal PC components, such as stairs, balconies and other complex PC components, it is difficult to summarise the design rules of the components from a large number of drawings and split them. Further, developing the corresponding tool software is difficult but worth exploring. The developed design approach has been successfully applied to vertical PC elements, but it can also be reused for other types of PC elements. Secondly, at present, some prefabricated projects are carried out by multiple parties, which makes the design and manufacturing disconnected and also causes the DFMA-oriented design pattern in this paper to not be well used. However, it can be well used in the general contracting mode of Architecture, Engineering and Construction (AEC). Finally, the DFMA-oriented design approach is applicable to different countries and regions due to the different design rules for assembled buildings. Structural design requirements need to be met differently, and the method needs to be adapted to suit local needs. However, the DFMA-oriented design concept and the secondary splitting modularity approach of this paper are applicable to all regions.

## Conclusion

In this paper, DFMA is introduced and a modular design approach that can cope with design diversity and achieve component and production standardization is proposed. The PC components as products are innovatively split into various standardised functional modules. Parametric design and BIM are combined, and the DFMA-oriented parametric design makes the vertical PC components highly assembleable and manufacturable. The established library of corresponding parametric modules enables standard modules to be formed into an infinite number of PC components, which are suitable for the front-end design and can match any house type, floor type and building height. Therefore, the design of PC components with fewer specifications and more combinations can be achieved, which solves the industry problem of the poor versatility of vertical PC components and lays the basis for the DFMA-oriented PC component design process. For manufacturing plants, PC components with a high standardization can match combined production moulds, reduce production difficulty, and improve mould reuse rate, thus achieving production standardization. The integrated application of BIM in the whole life cycle of prefabricated buildings is accelerated, which helps build a standardised design system for prefabricated buildings and improves the comprehensive benefits of prefabricated buildings.

The design, production and installation of assembled buildings are easy to disconnect and difficult to synchronise information and data. This paper proposes a DFMA-oriented way for the optimization of the whole process design of assembled buildings. The advantages of DFMA are fully exploited, and the design phase fully considers the needs of assembly. In the construction of the whole process life cycle, the cloud-based module library is continuously optimised through the verification of the production and installation process of PC components, making the subsequent production application more in line with the DFMA requirements and facilitating the construction of a standardised design system for assembled buildings.

## Data Availability

Some or all data, models, or code that support the findings of this study are available from the corresponding author upon reasonable request.

## References

[1] Jaillon L, Poon CS (2014). Life cycle design and prefabrication in buildings: A review and case studies in Hong Kong. Autom. Constr..

[2] Li Z, Shen GQ, Xue X (2014). Critical review of the research on the management of prefabricated construction. Habitat. Int..

[3] Polat G (2008). Factors affecting the use of precast concrete systems in the United States. J. Constr. Eng. Manag..

[4] Qi Y, Chang S, Ji Y, Qi K (2018). BIM-based incremental cost analysis method of prefabricated buildings in China. Sustainability.

[5] Liang N, Yu M (2021). Research on design optimization of prefabricated residential houses based on BIM technology. Sci. Program..

[6] Ma, G., Jiang, J. & Shang, S. Visualization of component status information of prefabricated concrete building based on building information modeling and radio frequency identification: A case study in China. *Adv. Civ. Eng.* **2019** (2019).

[7] Jaillon L, Poon CS, Chiang YH (2009). Quantifying the waste reduction potential of using prefabrication in building construction in Hong Kong. Waste Manag..

[8] Zhang X, Skitmore M, Peng Y (2014). Exploring the challenges to industrialized residential building in China. Habitat. Int..

[9] Li HX, Al-Hussein M, Lei Z, Ajweh Z (2013). Risk identification and assessment of modular construction utilizing fuzzy analytic hierarchy process (AHP) and simulation. Can. J. Civ. Eng..

[10] Nasereddin M, Mullens MA, Cope D (2007). Automated simulator development: A strategy for modeling modular housing production. Autom. Constr..

[11] Chan APC (2022). DfMA for a better industrialised building system. Buildings.

[12] Tam VWY, Tam CM, Zeng SX, Ng WCY (2007). Towards adoption of prefabrication in construction. Build. Environ..

[13] Kovacic I, Zoller V (2015). Building life cycle optimization tools for early design phases. Energy.

[14] Yuan Z, Sun C, Wang Y (2018). Design for manufacture and assembly-oriented parametric design of prefabricated buildings. Autom. Constr..

[15] Tan T, Mills G, Jiqiang H, Papadonikolaki E (2021). Integrated approaches to design for manufacture and assembly: A case study of huoshenshan hospital to combat COVID-19 in Wuhan, China. J. Manag. Eng..

[16] Choi JO (2020). Innovative technologies and management approaches for facility design standardization and modularization of capital projects. J. Manag. Eng..

[17] Ng LX, Wang ZB, Ong SK, Nee AYC (2013). Integrated product design and assembly planning in an augmented reality environment. Assem. Autom..

[18] Singh MM, Deb C, Geyer P (2022). Early-stage design support combining machine learning and building information modelling. Autom. Constr..

[19] Lu W (2020). Design for manufacture and assembly (DfMA) in construction: the old and the new. Arch. Eng. Des. Manag..

[20] Gao S, Jin R, Lu W (2019). Design for manufacture and assembly in construction: A review. Build. Res. Inf..

[21] Selvaraj P, Radhakrishnan P, Adithan M (2008). An integrated approach to design for manufacturing and assembly based on reduction of product development time and cost. Int. J. Adv. Manuf. Technol..

[22] Leminen V, Eskelinen H, Matthews S, Varis J (2013). Development and utilization of a DFMA-evaluation matrix for comparing the level of modularity and standardization in clamping systems. Mechanics.

[23] Bogue R (2012). Design for manufacture and assembly: Background, capabilities and applications. Assem. Autom..

[24] Tan T (2020). Construction-oriented design for manufacture and assembly guidelines. J. Constr. Eng. Manag..

[25] da Silva CES, Salgado EG, Mello CHP, da Silva Oliveira E, Leal F (2014). Integration of computer simulation in design for manufacturing and assembly. Int. J. Prod. Res..

[26] Fox S, Marsh L, Cockerham G (2010). Design for manufacture: A strategy for successful application to buildings. Constr. Manag. Econ..

[27] Al AMH, Hassanain MA, Juaim MN (2014). Evaluation and selection of curtain wall systems for medium-high rise building construction. Struct. Surv..

[28] Chen K, Lu W (2018). Design for manufacture and assembly oriented design approach to a curtain wall system: A case study of a commercial building in Wuhan, China. Sustainability.

[29] Gbadamosi AQ (2020). Big data for design options repository: Towards a DFMA approach for offsite construction. Autom. Constr..

[30] Bynum P (2012). Building information modeling in support of sustainable design and construction. J. Constr. Eng. Manag..

[31] Giel BK, Issa RRA, Asce F (2011). Return on investment analysis of using building information modeling in construction. J. Comput. Civ. Eng..

[32] Li CZ, Xue F, Li X, Hong J, Shen GQ (2018). An internet of things-enabled BIM platform for on-site assembly services in prefabricated construction. Autom. Constr..

[33] Cao D (2015). Practices and effectiveness of building information modelling in construction projects in China. Autom. Constr..

[34] Fadeyi MO (2017). The role of building information modeling (BIM) in delivering the sustainable building value. Int. J. Sustain. Built Environ..

[35] Park J (2018). BIM-based parametric design methodology for modernized Korean traditional buildings. J. Asian Arch. Build. Eng..

[36] Oxman R (2017). Thinking difference: Theories and models of parametric design thinking. Des. Stud..

[37] Wortmann T, Tunçer B (2017). Differentiating parametric design: Digital workflows in contemporary architecture and construction. Des. Stud..

[38] Bryde D, Broquetas M, Volm JM (2013). The project benefits of building information modelling (BIM). Int. J. Project Manag..

[39] Chen K (2018). A physical internet-enabled building information modelling system for prefabricated construction. Int. J. Comput. Integr. Manuf..

[40] Nath T, Attarzadeh M, Tiong RLK, Chidambaram C, Yu Z (2015). Productivity improvement of precast shop drawings generation through BIM-based process re-engineering. Autom. Constr..

[41] Isaac S, Bock T, Stoliar Y (2016). A methodology for the optimal modularization of building design. Autom Constr.

[42] Bai S, Li M, Asce AM, Lingguang S, Kong R (2020). Developing a common library of prefabricated structure components through graphic media mapping to improve design efficiency. J. Constr. Eng. Manag..

[43] Li J (2018). Design and climate-responsiveness performance evaluation of an integrated envelope for modular prefabricated buildings. Adv. Mater. Sci. Eng..

[44] Cao J, Bucher DF, Hall DM, Eggers M (2022). A graph-based approach for module library development in industrialized construction. Comput. Ind..

[45] Martínez S, Jardón A, Víctores JG, Balaguer C (2013). Flexible field factory for construction industry. Assem. Autom..

[46] Knippers J (2013). From model thinking to process design. Archit. Des..

[47] Kim JB, Jeong W, Clayton MJ, Haberl JS, Yan W (2015). Developing a physical BIM library for building thermal energy simulation. Autom. Constr..

[48] Kim S, Nussbaum MA, Jia B (2012). The benefits of an additional worker are task-dependent: Assessing low-back injury risks during prefabricated (panelized) wall construction. Appl. Ergon..

[49] Kim MK, Cheng JCP, Sohn H, Chang CC (2015). A framework for dimensional and surface quality assessment of precast concrete elements using BIM and 3D laser scanning. Autom. Constr..

[50] Mao C (2016). Cost analysis for sustainable off-site construction based on a multiple-case study in China. Habitat. Int..

[51] Qi Y, Chang S, Ji Y, Qi K (2018). BIM-based incremental cost analysis method of prefabricated buildings in China. Sustainability (Switzerland).

[52] Zhang W, Lee MW, Jaillon L, Poon CS (2018). The hindrance to using prefabrication in Hong Kong’s building industry. J. Clean Prod..

[53] Jaillon L, Poon CS (2010). Design issues of using prefabrication in Hong Kong building construction. Constr. Manag. Econ..

